# Osteoporosis as a Potential Modifiable Risk Factor for Dementia in Thailand: A Cross-Sectional Analysis

**DOI:** 10.7759/cureus.63511

**Published:** 2024-06-30

**Authors:** Parit Hiri-O-tappa, Kasidid Lawongsa, Supatcha Kengpanich, Patsri Srisuwan

**Affiliations:** 1 Family Medicine, Phramongkutklao Hospital, Bangkok, THA; 2 Geriatrics, Phramongkutklao Hospital, Bangkok, THA

**Keywords:** public health, cognitive impairment, risk factors, dementia, osteoporosis

## Abstract

Objective: This study aims to explore the potential of osteoporosis as a modifiable risk factor for dementia and investigate whether treatments like estrogen supplementation and bisphosphonates can reduce the risk of dementia in the Thai population. This study aimed to evaluate the risk of dementia in patients with osteoporosis.

Materials and methods: A cross-sectional analysis was conducted. Participants aged <50 years and those with a history of dementia at baseline were excluded. Clinical variables were analyzed using the chi-squared test. Logistic regression analysis was conducted to determine independent factors of dementia after adjusting for potential confounders.

Results: The cohort was conducted on 54,399 participants, of whom 9763 (17.9%) had osteoporosis. The results indicated that individuals with osteoporosis had an odds ratio (OR) of 1.62-fold higher risk of developing dementia compared to those without osteoporosis (95% confidence interval (CI) 1.37-1.92, p-value < 0.001). Furthermore, compared to patients with osteoporosis not undergoing any treatment, those receiving estrogen supplementation and bisphosphonate treatment showed reduced associations with dementia, with an OR of 0.84 (95% CI 0.73-0.98, p-value = 0.023) and 0.66 (95% CI 0.60-0.73, p-value < 0.001), respectively.

Conclusion: The study findings suggest an elevated risk of dementia in patients with osteoporosis and contribute substantially to our understanding of the link between osteoporosis and dementia in the Thai population.

## Introduction

Osteoporosis presents a formidable health challenge, especially among older adults, and is characterized by diminished bone density and increased fracture susceptibility [1]. While fractures primarily affect physical health, recent research suggests a potential connection between osteoporosis and cognitive decline, including dementia [2]. The convergence of skeletal health and cognitive function highlights the need to understand the broad implications [3].

With advancing age, individuals face increased vulnerability to both osteoporosis and dementia, which significantly affect their quality of life and independence. Osteoporotic fractures not only cause pain and physical limitations but also trigger a cascade of adverse outcomes, including prolonged hospital stays, decreased mobility, and an increased risk of mortality. Similarly, dementia, a progressive neurodegenerative condition, compromises cognitive faculties, memory, and behavior, often necessitating substantial caregiving and medical intervention [4].

Although osteoporosis and dementia may appear distinct, recent studies have revealed shared risk factors and potential mechanistic links. Factors such as age, sex, hormonal imbalances, and genetic predispositions underscore the complex interplay between skeletal health and cognitive function. Additionally, emerging evidence suggests that chronic inflammation, vascular dysfunction, and alterations in hormonal signaling are potential contributors to the pathogenesis of the two conditions [5].

Despite intriguing associations, the precise nature of the osteoporosis-dementia relationship remains elusive [6]. While some studies posit that osteoporosis is an early indicator or risk factor of cognitive decline, others propose bidirectional causality or shared underlying pathophysiological mechanisms [7]. Therefore, unraveling the complex interplay between skeletal health and cognitive function is crucial for devising holistic strategies to address the burden of osteoporosis and dementia in aging populations.

Given the increasing global prevalence of osteoporosis and dementia, further research is imperative to elucidate their association and explore therapeutic interventions. Integrating multidisciplinary approaches from geriatrics, neurology, endocrinology, and epidemiology can improve our understanding of the underlying mechanisms linking osteoporosis and dementia and pave the way for targeted interventions to foster healthy aging and preserve cognitive function.

## Materials and methods

Data source and study population

The Phramongkutklao Hospital Database (PHD) program at Phramongkutklao Hospital in Bangkok, Thailand, is a comprehensive database system designed to provide access to patient information. Our study utilized a specific subset of this database, which contained detailed healthcare records from the outpatient department that were randomly selected across all patients seeking care. These datasets are noteworthy for their ability to be linked using encrypted and unique personal identification numbers, allowing the creation of longitudinal medical histories for each patient. The diagnostic classification followed the guidelines of the International Classification of Diseases, 10th Revision, and Clinical Modification (ICD-10-CM) [8]. This study was approved by the Institutional Review Board of the Royal Thai Army Medical Department (IRBTA0275/2567).

Study patients

**Figure 1:**
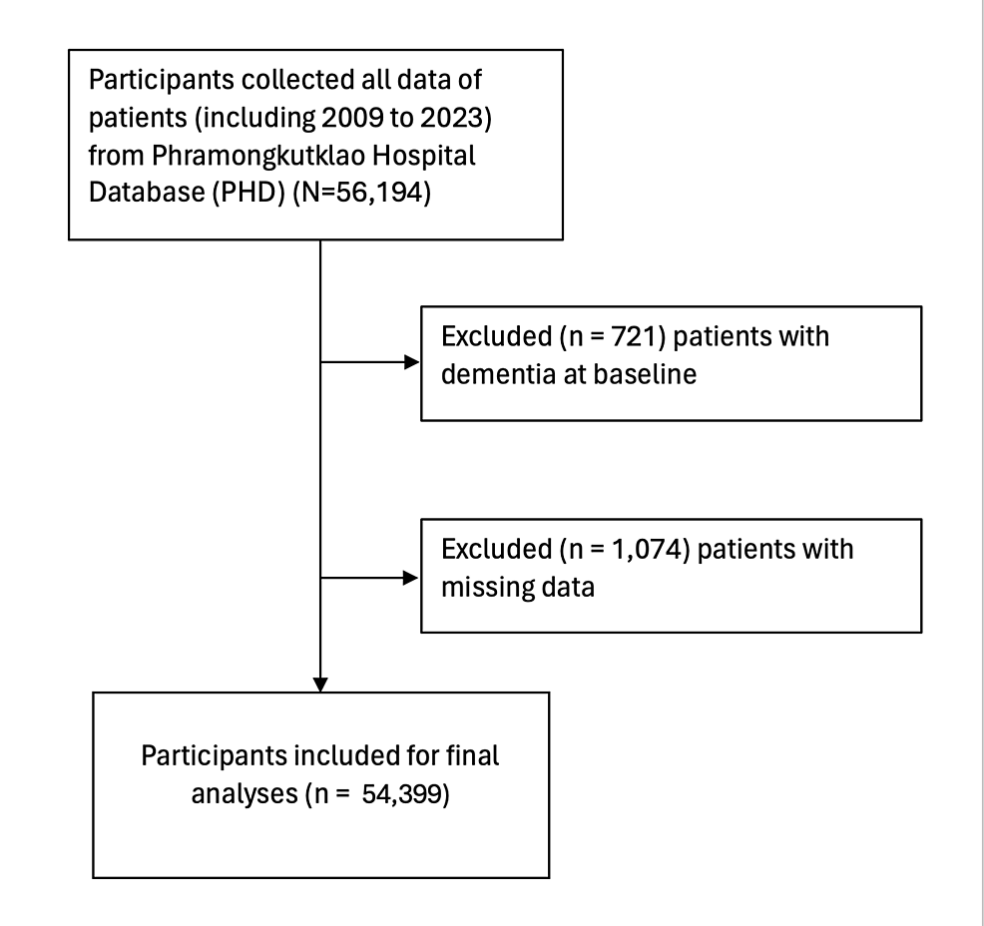
Flowchart for participants with bone mineral density scans included in the study

A cross-sectional analysis was performed to assess the association between osteoporosis and osteoporotic fractures (ICD-10-CM codes: M80-M82) and dementia (ICD-10-CM codes: F00-F03, G30). Patients with osteoporotic fractures were included in the cohort due to the underdiagnosis of osteoporosis [9]. The study included patients diagnosed with new-onset osteoporosis between 2009 and 2023, including those identified at their first diagnosis. Exclusion criteria were age under 50 years and a history of dementia at baseline. We gathered patient data from 2009 to 2023 from the PHD as a comparison cohort, applying the same exclusion criteria. In total, 54,399 participants were included, with 9763 (17.9%) having osteoporosis. All data were collected until December 31, 2023, or until they were lost to follow-up, died, or transferred to another hospital. At baseline, between 2009 and 2023, the cohorts included 54,399 participants from the PHD. Patients with dementia at baseline (n = 721) and those with missing data (n = 1,074) were excluded. Ultimately, 2214 (4.1%) people with dementia and 9763 (17.9%) with osteoporosis before dementia were included in the study (Figure 1).

Definitions

The variables considered in the study included age (≥50 years), sex (male or female), weight, height, and income. Baseline comorbidities were identified using ICD-10-CM codes and anatomical therapeutic chemical (ATC) codes for medications. Comorbidities included type 2 diabetes (ICD-10-CM code: E11), hypertension (ICD-10 codes: I10-I15), stroke (ICD-10-CM codes: I60-I66), depression (ICD-10-CM codes: F32-F33), estrogen supplementation (ATC codes: T385, Y425, EST103N, PRO108E, ANG103N), and bisphosphonate use (ATC codes: ALE104E, FOS103N, BON103N, ACT107N, RIS108N, ACL201N, ZOL203N, ZOL202N, PRO213N, XGE200N, CEL104N, MIA201N, FOR202N, EVE201N), a primary treatment for osteoporosis. The control group with osteoporotic risk was classified into low-, intermediate-, and high-risk osteoporosis groups using the osteoporosis self-assessment tool.

Osteoporosis

The diagnosis of osteoporosis was the dual-energy X-ray absorptiometry (DXA or DEXA) scan, which is typically conducted at the hip and spine. The results were reported as T-scores, with a T-score of -2.5 or lower indicating osteoporosis, suggesting a bone density below the expected range for age.

Dementia

Diagnosing dementia involves the mini-mental state examination (MMSE) to assess cognitive function, and confirmation is often achieved through magnetic resonance imaging (MRI) or computed tomography (CT) scans to detect structural brain changes, such as atrophy, strokes, tumors, or other abnormalities.

Statistical analysis

All analyses were conducted using IBM SPSS Statistics software (version 26.0; IBM Corp., Armonk, NY). The relationships between the variables and osteoporosis were assessed using the chi-square test. Demographic data and dementia diagnoses were compared between the two groups. Clinical variables were analyzed using the chi-square test. Logistic regression analysis was performed to identify independent factors associated with dementia, adjusting for potential confounders such as age, sex, BMI, income, type 2 diabetes, hypertension, stroke, depression, estrogen supplementation, and bisphosphonate use. To prevent variable selection due to multicollinearity correlations, only variables with a p-value of less than 0.1 in univariate analysis were included in the multivariate logistic regression model.

## Results

Demographic characteristics

This cross-sectional analysis utilized data from 54,399 patients, including 25,181 (46.3%) males, 35,683 (65.6%) elderly (age > 65), and 2,951 (5.4%) underweight (BMI < 18.5) patients. Table 1 shows the demographic characteristics and baseline comorbidity statuses of the patients. Among the included patients, 9,763 (17.9%) had osteoporosis. Moreover, 26,805 (49.3%) fell into the high-risk group of osteoporosis, 16,183 (29.7%) fell into the intermediate-risk group, and the risk prevalence differed significantly (p < 0.001). The prevalence rate of dementia was 2,214(4.1%), and this significantly differed between sexes (p = 0.009). The prevalence of no income was 46,075 (84.7%), hypertension was 50,157 (92.2%), stroke was 3,659 (6.7%), depression was 2,006 (3.7%), and bisphosphonate usage was 7,203 (13.2%), and the prevalence of dementia significantly differed between these (p < 0.001).

**Table 1:** Comparison of baseline characteristics between patients with and without dementia Note: Values are shown as mean ± SD or number of participants (%). BMI: Body mass index; OSTA: Osteoporosis self-assessment tool for Asians. *p-value < 0.001; ** p-value < 0.01.

Variable (units)		Dementia		Total n (%)	p-value
		No, n (%)	Yes, n (%)		
Total		52,185 (95.9)	2,214 (4.1)	54,399	-
Sex	Male	24,216 (96.2)	965 (3.8)	25,181 (46.3)	0.009**
	Female	27,969 (95.7)	1,249 (4.3)	29,218 (53.7)	-
Age (years)	≤65	18,597 (99.4)	119 (0.6)	18,716 (34.4)	<0.001*
	>65	33,588 (94.1)	2,095 (5.9)	35,683 (65.6)	-
BMI (kg/m^2^)	<18.5	2,696 (91.4)	255 (8.6)	2,951 (5.4)	<0.001*
	≥18.5	49,488 (96.2)	1,959 (3.8)	51,447 (94.6)	-
Income	No income	43,897 (95.3)	2,178 (4.7)	46,075 (84.7)	<0.001*
	Income	8,288 (99.6)	36 (0.4)	8,324 (15.3)	-
OSTA	Low	11,312 (99.1)	99 (0.9)	11,411 (21.0)	<0.001*
	Intermediate	15,862 (98.0)	321 (2.0)	16,183 (29.7)	-
	High	25,011 (93.3)	1,794 (6.7)	26,805 (49.3)	-
Osteoporosis	No	43,090 (96.5)	1,546 (3.5)	44,636 (82.1)	<0.001*
	Yes	9,095 (93.2)	668 (6.8)	9,763 (17.9)	-
Type 2 diabetes	No	49,753 (95.9)	2,125 (4.1)	51,878 (95.4)	0.160
	Yes	2,432 (96.5)	89 (3.5)	2,521 (4.6)	-
Hypertension	No	3,948 (93.1)	294 (6.9)	4,242 (7.8)	<0.001*
	Yes	48,237 (96.2)	1,920 (3.8)	50,157 (92.2)	-
Stroke	No	48,960 (96.5)	1,780 (3.5)	50,740 (93.3)	<0.001*
	Yes	3,225 (88.1)	434 (11.9)	3,659 (6.7)	-
Depression	No	50,437 (96.3)	1,956 (3.7)	52,393 (96.3)	<0.001*
	Yes	1,748 (87.1)	258 (12.9)	2,006 (3.7)	-
Estrogen supplementation	No	51,798 (95.9)	2,200 (4.1)	53,998 (99.3)	0.556
	Yes	387 (96.5)	14 (3.5)	401 (0.7)	-
Bisphosphonate	No	45,528 (96.5)	1,668 (3.5)	47,196 (86.8)	<0.001*
	Yes	6,657 (92.4)	546 (7.6)	7,203 (13.2)	-

Association of osteoporosis and dementia

Table 2 displays the association between osteoporosis and dementia. An unadjusted analysis revealed that the OR for dementia was 2.05 in patients with osteoporosis (95% CI 1.86-2.25, p < 0.001). For those in the high-risk osteoporosis group, the OR was 8.2 (95% CI 6.69-10.05, p < 0.001), while the intermediate-risk group had an OR of 2.3 (95% CI 1.84-2.90, p < 0.001). After adjusting for age, sex, income, BMI, and other confounding factors such as hypertension, stroke, and depression, the OR was 1.62 (95% CI 1.37-1.92, p < 0.001). For the high-risk osteoporosis group, the adjusted OR was 3.49 (95% CI 2.99-4.08, p < 0.001), and for the intermediate-risk group, it was 1.78 (95% CI 1.53-2.07, p < 0.001). Other risk factors that increased the likelihood of dementia included being female (OR 1.22, 95% CI 1.12-1.34), being above 65 years of age (OR 5.05, 95% CI 4.15-6.16), having no income (OR 1.83, 95% CI 1.28-2.60), having hypertension (OR 2.19, 95% CI 1.94-2.47), having a history of stroke (OR 3.22, 95% CI 2.90-3.58), and experiencing depression (OR 3.44, 95% CI 3.00-3.95), compared to those without these risk factors.

**Table 2:** Univariate and multivariate analysis for association between osteoporosis, other factors, and dementia ^a^Multiple analyses included age, sex, income, BMI, OSTA, osteoporosis, hypertension, stroke, and depression. *p-value < 0.001; ** p-value < 0.01. BMI: Body mass index; OSTA: Osteoporosis self-assessment tool for Asians.

Variable (units)		Crude OR (95% CI)	p-value	Adjusted^a^ OR (95% CI)	p-value
Sex	Female	1.12 (1.03-1.22)	0.009**	1.22 (1.12-1.34)	<0.001*
Age (years)	>65	9.75 (8.10-11.74)	<0.001*	5.05 (4.15-6.16)	<0.001*
BMI (kg/m^2^)	<18.5	2.39 (2.09-2.74)	<0.001*	1.02 (0.93-1.13)	0.658
Income	No	11.42 (8.21-15.89)	<0.001*	1.83 (1.28-2.60)	<0.001*
OSTA	Low	Ref.			
	Intermediate	2.31 (1.84-2.90)	<0.001*	1.78 (1.53-2.07)	<0.001*
	High	8.20 (6.69-10.05)	<0.001*	3.49 (2.99-4.08)	<0.001*
Osteoporosis		2.05 (1.86-2.25)	<0.001*	1.62 (1.37-1.92)	<0.001*
Type 2 diabetes		1.17 (0.94-1.45)	0.161	-	-
Hypertension		1.87 (1.65-2.12)	<0.001*	2.19 (1.94-2.47)	<0.001*
Stroke		3.70 (3.31-4.14)	<0.001*	3.22 (2.90-3.58)	<0.001*
Depression		3.81 (3.31-4.37)	<0.001*	3.44 (3.00-3.95)	<0.001*

Table 3 shows that, when analyzing the effects of medication on dementia in all patients with osteoporosis, those receiving estrogen supplementation and bisphosphonate treatment had 0.84-fold (p = 0.023) and 0.66-fold (p < 0.001) lower associations with dementia, respectively, compared to patients with osteoporosis who did not receive any treatment.

**Table 3:** Odds ratio of dementia in all osteoporosis patients by treatment ^a^Multiple analyses included age, sex, income, BMI, OSTA, osteoporosis, hypertension, stroke, and depression. *p-value < 0.001; **p-value < 0.05. OR: Odds ratio.

Osteoporosis patients	Crude OR (95% CI)	p-value	Adjusted^a^ OR (95% CI)	p-value
Without treatment	Ref.			
With treatment				
Estrogen supplementation	0.45 (0.40-0.49)	<0.001*	0.84 (0.73-0.98)	0.023**
Bisphosphonate	0.48 (0.43-0.53)	<0.001*	0.66 (0.60-0.73)	<0.001*

## Discussion

Our study is one of the few investigations into the risk of dementia among patients with osteoporosis in Thailand. We found a significantly higher risk for dementia in individuals with osteoporosis than in those without. Specifically, individuals with osteoporosis had an OR of 1.62 for developing dementia compared to those without osteoporosis (95% CI 1.37-1.92, p < 0.001). Furthermore, the results showed that osteoporosis treatment had a significant impact on the development of dementia. Patients with osteoporosis who received estrogen supplementation had a reduced risk of developing dementia with an OR of 0.84 (95% CI 0.73-0.98, p = 0.023). Similarly, those receiving bisphosphonate treatment also showed a reduced risk, with an OR of 0.66 (95% CI 0.60-0.73, p < 0.001). These observations underscore the importance of recognizing osteoporosis not only as a skeletal disorder but also as a potential contributor to cognitive impairment, and they highlight the potential benefits of osteoporosis treatments in reducing the risk of dementia [10].

The observed link between osteoporosis and dementia is consistent with previous findings from global studies, highlighting the complex interplay between skeletal health and cognitive function [11-13]. Although the precise mechanisms remain unclear, shared risk factors, such as age, sex, hormonal imbalances, and genetic predispositions, may play a role. Additionally, factors like chronic inflammation, vascular dysfunction, and hormonal signaling alterations may mediate the relationship between osteoporosis and dementia [14].

The theory that osteoporosis is a risk factor for dementia suggests that the deterioration of bone health may be associated with cognitive decline. This association is grounded in several potential shared mechanisms and risk factors. First, both osteoporosis and dementia are influenced by aging, hormonal changes, and lifestyle factors such as physical inactivity, poor nutrition, and smoking. Second, vascular health plays a crucial role in both conditions. Compromised blood flow and vascular damage, which are common in osteoporosis, can also lead to reduced brain perfusion and contribute to cognitive decline. Third, chronic systemic inflammation is implicated in both osteoporosis and neurodegeneration. Elevated inflammatory markers, such as C-reactive protein (CRP) and interleukins, can damage both bone tissue and neural cells. Additionally, low levels of vitamin D, which is essential for bone health, have been associated with an increased risk of dementia. The bidirectional nature of this relationship suggests that interventions aimed at improving bone health might also have beneficial effects on cognitive function, highlighting the importance of comprehensive health management in older adults [15-17].

Previous research undertaken [7] indicated a negative association between estrogen levels and the risk of osteoporosis or dementia. Jilka et al. [18] elucidated that decreased estrogen levels stimulate osteoclast development. Our investigation into the impact of estrogen supplementation among patients with osteoporosis revealed a reduced risk of dementia among those receiving estrogen supplementation compared to untreated individuals. Nevertheless, the point that not all individuals receiving estrogen supplementation necessarily have osteoporosis is noteworthy.

They hypothesize that anti-osteoporosis agents might reduce the occurrence of dementia based on several key factors. First, estrogen supplementation, commonly used to treat osteoporosis, has demonstrated neuroprotective effects through its action on cholinergic neurons, providing neurotrophic, antioxidant, and anti-inflammatory benefits [19,20]. Previous studies have shown that hormone or estrogen therapy can reduce the incidence or delay the onset of dementia, particularly Alzheimer’s disease [21,22]. Second, bisphosphonates, which are used to treat osteoporosis by inducing osteoclast apoptosis and improving bone health, may indirectly influence overall health and reduce the risk of dementia. The study’s findings indicate a negative correlation between osteoporosis treatment (both bisphosphonates and estrogen supplementation) and dementia risk among patients with osteoporosis. This correlation suggests that treating osteoporosis may have protective effects against the development of dementia. Furthermore, previous research indicates that patients with dementia are less likely to receive osteoporosis medications like bisphosphonates, implying that untreated osteoporosis could contribute to higher dementia risk, and thus, proper treatment might mitigate this risk [23]. Additionally, effective management of osteoporosis can improve a patient’s overall mobility and physical health, potentially reducing the risk of falls and related complications, which are associated with cognitive decline and dementia. Therefore, these points collectively support the hypothesis that anti-osteoporosis agents could play a role in reducing the occurrence of dementia.

Studying their link is crucial for several reasons. First, these conditions share common risk factors such as aging, hormonal changes, and lifestyle factors (e.g., physical inactivity and poor nutrition), which can provide insights into comprehensive prevention strategies that address both conditions simultaneously [14]. Second, the bidirectional impact suggests that treating one condition could potentially influence the management of the other. For instance, osteoporosis treatment can improve mobility and reduce fall risk, which might positively affect cognitive health. Third, recognizing the association can lead to better clinical practices, where patients diagnosed with osteoporosis are monitored more closely for cognitive decline, enabling earlier detection and intervention for dementia [24]. Lastly, understanding this link promotes holistic patient care, ensuring that interventions are not siloed and the overall health and well-being of the patient are considered.

The study findings have implications for public health interventions and policies. Early identification of patients with osteoporosis at risk of dementia could lead to targeted screening and prevention strategies to preserve cognitive function and improve overall quality of life. Collaboration across disciplines such as geriatrics, neurology, endocrinology, and epidemiology is essential to comprehensively address the complex interrelationships between osteoporosis and dementia.

Limitations

This study has some limitations that should be considered. The PHD lacks detailed data on infections, brain injuries, substance abuse, metabolic disorders, chronic inflammatory diseases, tumors, toxic exposures, nutritional deficiencies, and severe psychiatric conditions, all of which may be potential risk factors for dementia [25]. Moreover, there is a lack of longitudinal data to track the progression of both osteoporosis and dementia over time. While cross-sectional data can provide a snapshot of the association between these conditions, it does not capture the temporal sequence or the dynamic nature of their progression. Longitudinal studies are needed to better understand the causal relationship between osteoporosis treatment and the onset or progression of dementia. Without long-term follow-up, it is challenging to determine whether the observed associations are due to direct effects of the treatments, shared risk factors, or other confounding variables that may change over time.

## Conclusions

Our study is a pioneering investigation of the association between osteoporosis and dementia in Thai individuals. We have revealed a substantial link between osteoporosis and an increased risk of dementia, emphasizing the necessity of perceiving osteoporosis not solely as a skeletal ailment but also as a potential predictor or influencer of cognitive decline. The implications of this study extend beyond clinical practice to public health interventions and policy considerations. Early detection of osteoporosis in patients with an elevated risk of dementia could trigger tailored screening, prevention, and treatment approaches aimed at safeguarding cognitive function and improving overall well-being. Collaborative efforts among health care professionals from various disciplines are imperative to comprehensively address the intricate connection between osteoporosis and dementia.
